# Gluteal Complex is important in External Snapping Hip: intraoperative identification of syndrome origin and endoscopic stepwise release–a case series.

**DOI:** 10.1007/s00264-023-05961-0

**Published:** 2023-09-05

**Authors:** Konrad Malinowski, Marcin Mostowy, Dong Woon Kim, Michalina Bawor, Paweł Skowronek, Michael T. Hirschmann, Przemysław A. Pękala, Robert F. LaPrade

**Affiliations:** 1 Department of Anatomy, Jagiellonian University Medical College, International Evidence-Based Anatomy Working Group, Kraków, Poland; 2 Artromedical Orthopedic Clinic, Antracytowa 1, 97-400 Bełchatów, Poland; 3 Orthopedic and Trauma Department, Veteran’s Memorial Teaching Hospital in Lodz, Medical University of Lodz, Żeromskiego 113, 90-549, Lodz, Poland; 4 Medical University of Lodz, Lodz, Poland; 5 Department of Orthopaedic and Trauma Surgery S. Żeromski Hospital, Os. Na Skarpie 66, 31-913 Kraków, Poland; 6 Department of Orthopaedic Surgery and Traumatology, Kantonsspital Baselland (Bruderholz, Liestal, Laufen), CH-4101 Bruderholz, Switzerland University of Basel, CH-4051 Basel, Switzerland; 7 Faculty of Medicine and Health Sciences, Andrzej Frycz Modrzewski Kraków University, Kraków, Poland; 8 Twin Cities Orthopedics, Edina, Minnesota USA

**Keywords:** External snapping hip, Iliotibial band, Gluteus maximus complex, Endoscopy, Release, Stepwise

## Abstract

**Purpose:**

External snapping hip syndrome (ESHS) was historically attributed to isolated iliotibial band (ITB) contracture. However, the gluteus maximus complex (GMC) may also be involved. This study aimed to intraoperatively identify the ESHS origin and assess the outcomes of endoscopic treatment based on the identified aetiological type.

**Methods:**

From 2008-2014, 30 consecutive patients (34 hips) with symptomatic ESHS cases refractory to conservative treatment underwent endoscopic stepwise “fan-like” release, gradually addressing all known reasons of ESHS: from the isolated ITB, through the fascial part of the GMC until a partial release of gluteus maximus femoral attachment occurred. Snapping was assessed intra-operatively after each surgical step and prospectively recorded. Functional outcomes were assessed via the MAHORN Hip Outcome Tool (MHOT-14).

**Results:**

Twenty seven patients (31 hips) were available to follow-up at 24-56 months. In all cases, complete snapping resolution was achieved intra-operatively: in seven cases (22.6%) after isolated ITB release, in 22 cases (70.9%), after release of ITB + fascial part of the GMC, and in two cases (6.5%) after ITB + fascial GMC release + partial release of GM femoral insertion. At follow-up, there were no snapping recurrences and MHOT-14 score significantly increased from a pre-operative average of 46 to 93(*p*<0.001).

**Conclusion:**

Intraoperative identification and gradual addressing of all known causes of ESHS allows for maximum preservation of surrounding tissue during surgery while precisely targeting the directly involved structures. Endoscopic stepwise “fan-like” release of the ITB and GMC is an effective, tailor-made treatment option for ESHS regardless of the snapping origin in the patients with possibility to manually reproduce the snapping.

**Supplementary Information:**

The online version contains supplementary material available at 10.1007/s00264-023-05961-0.

## Introduction

External snapping hip syndrome (ESHS), also known as coxa saltans externa, has been reported to be caused by the snapping of a thickened posterior portion of the iliotibial band (ITB) or the anterior part of the gluteus maximus (GM) complex (GMC) over the greater trochanter during hip motion [1–5].

Various surgical techniques for the treatment of ESHS have been reported in the literature, including Z- or N-plasty lengthening of the ITB [2, 6, 7], release or resection of a portion of the ITB [2, 8–12], and release of the GM femoral insertion [1, 13, 14], all of which can be performed via either an open or endoscopic approach. A review by Randelli et al. reported endoscopic techniques, as compared to open surgery to have fewer complications, lower recurrence rates, and better clinical and cosmesis [15]. On the other hand, recent systematic review by Sugrañes et al., does not seem to prioritise endoscopic techniques in ESHS treatment [16]. Regardless of the chosen surgical modality, recent literature reviews of surgical techniques for ESHS treatment present that they mainly centre around relieving the tension of the ITB and are not focused on the identification of which anatomical structures are responsible for snapping in a particular patient [7, 17]. However, recent studies have demonstrated that ESHS may be caused not only by a tensed ITB, but also by structures of the GMC, such as the gluteal fascia and the proximal portion of the GM femoral insertion [3, 7, 13, 18]. This has led to the development of surgical techniques tailored to the stepwise localisation of contracted tissues [7, 19, 20].

We hypothesised that the intraoperative identification and gradual addressing of all known causes of ESHS would allow to safely eliminate snapping in all patients. Within this hypothesis, the gradual addressing of ESHS was defined as an assessment if snapping resolved after each surgical step. The first step was an isolated ITB release, the second step was to release a fascial part of the GMC and the third step was to release the proximal part of GM femoral insertion. Therefore, the aim of this study was to intraoperatively identify the origins of ESHS and to assess the outcomes of endoscopic treatment based on the identified aetiological type.

## Materials and methods

Ethical approval was obtained from the district medical chamber (approval number K.B.-7/2023). The study was designed in compliance with the Helsinki Declaration and informed consent was obtained from each participant.

### Patients

**Fig. 1:**
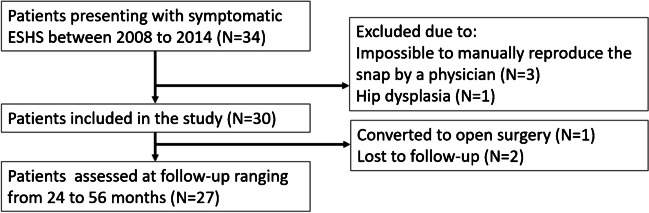
Flowchart of patients screening and inclusion. ESHS, External Snapping Hip Syndrome

This was a retrospective cohort study of consecutive patients presenting to a single centre with symptomatic cases of ESHS between 2008 and 2014. All data were prospectively collected into a database. Inclusion criteria were: 1) symptomatic ESHS, refractory to conservative treatment, 2) the ability to manually and passively reproduce the snap on clinical examination and under anaesthesia, which was necessary for the intra-operative assessment. Exclusion criteria were: (1) hip dysplasia, (2) prior surgeries to the hip and/or associated areas, (3) “mega-trochanter”, (4) Trendelenburg sign and/or signs of gluteus medius or gluteus minimus tears on magnetic resonance imaging (MRI), (5) general laxity disorders. From 34 consecutive patients who presented with symptomatic ESHS, 30 patients (18 to 32 years old, 26 female, four male) underwent the endoscopic stepwise “fan-like” release of the ITB and GMC and were included in this study (Fig. 1). Twenty six patients presented with unilateral snapping, while four presented it bilaterally, for a total of 34 hips. Among 30 patients (34 hips) included in the study, one male patient was converted to open surgery and two female patients were lost prior to final follow-up, resulting in 27 patients (31 hips - 91.1%) available at follow-up ranging from 24 to 56 months (Fig. 1).

### Surgical technique

**Fig. 2:**
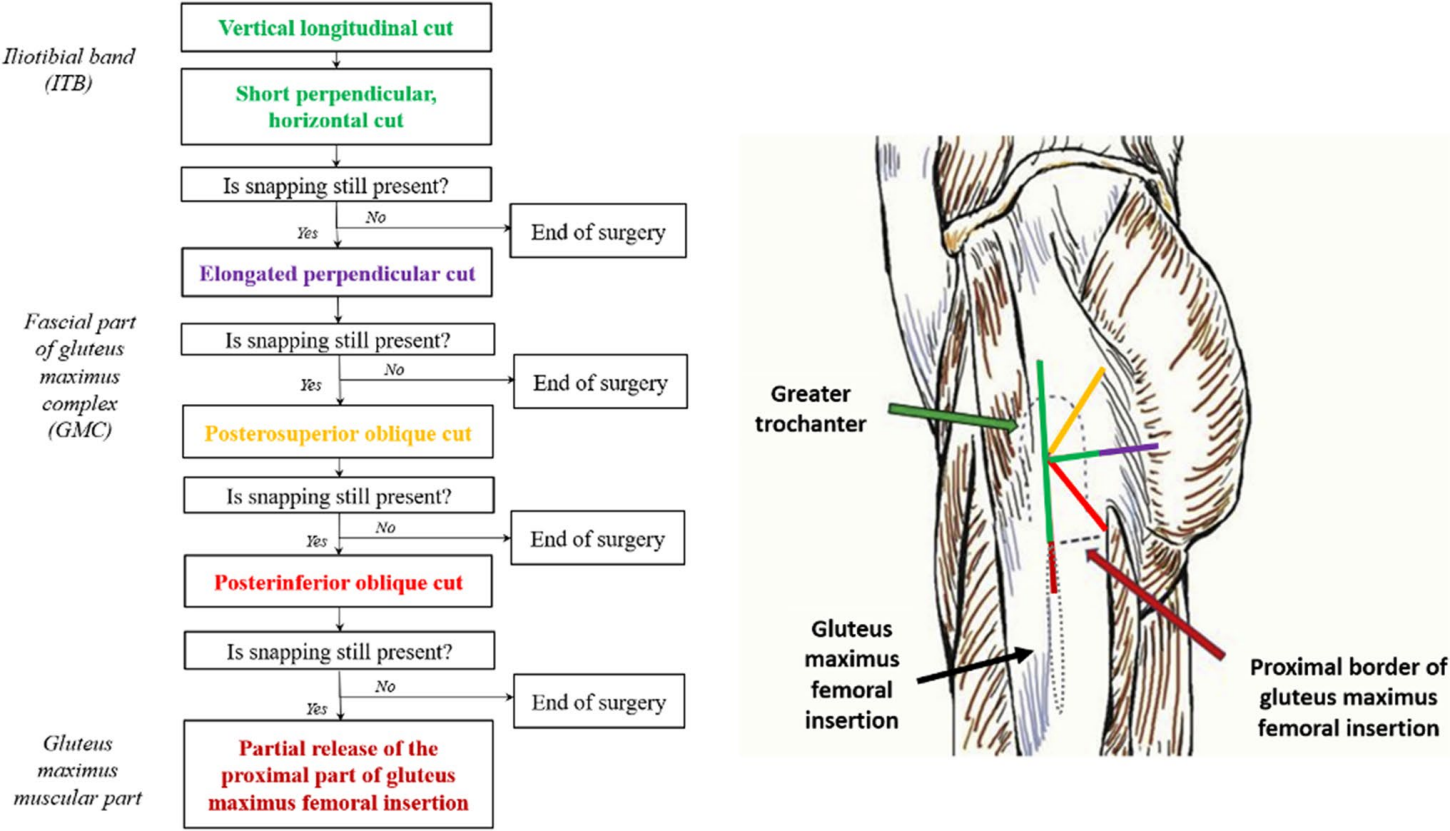
Schematic representation of the technique. Reproduced with modifications from Malinowski et al. [7]

All patients were treated by endoscopic stepwise “fan-like” release of the ITB and GMC previously described in literature [7]. The procedure was performed with the patient under spinal or general anaesthesia in the lateral decubitus position. The operated leg was draped in a sterile fashion, allowing full hip range of motion (ROM), so that the surgeon can freely reproduce the snapping intraoperatively. It is very important to reach the end range of hip flexion and internal rotation during snapping assessment, as depending on the most contracted tissues. The snapping can be present in slight hip flexion or on contrary, near the full hip flexion close to the chest. The proximal to distal level where snapping could be palpated most clearly was determined and marked on the skin. Usually this was the level most prominent part of the greater trochanter. An inferior trochanteric portal (ITP) was made three to five centimetres distal to this mark by a stab incision. The working space for arthroscopic instruments was created by blunt dissection over the surface of ITB through the ITP. Next, a superior trochanteric (STP) portal was made under a direct visualisation three to five centimetres proximal to the previously marked level and was further used as the working portal for arthroscopic instruments. A shaver, introduced through the STP, was used to gently remove connective fibres between the subcutaneous tissue and the ITB. Meticulous haemostasis and operative field widening were achieved using radiofrequency probe. In full hip extension, a longitudinal incision of the ITB was made along the natural course of its fibres with the radiofrequency probe, starting at the vastus tubercle level. The incision was extended proximally to the upper border of the greater trochanter till the muscle fibres of the tensor fasciae latae and distally to the point of GM femoral insertion (longitudinal green line on Fig. 2). Afterwards, a posteriorly directed incision, perpendicular to the previously made longitudinal one, was started at the level of the most contracted tissues. It was extended towards the posterior trochanteric facet and posterior trochanteric crest until they were clearly visible, and the “red” anterior muscular tissue of the GM muscle belly was reached (perpendicular green line in Fig. 2). All tissues were released all the way to the trochanteric gluteal tendons insertion - even thin inflammatory membranes cannot be left uncut. The cut was only as wide as the width of radiofrequency probe and no tissue was excised. At this point, the presence of snapping was assessed for the first time. If it was absent, the surgery was stopped and the ITB was considered to be the only tissue responsible for the occurrence of symptoms in these particular patient. In cases where the described above longitudinal and “short” perpendicular incisions had not eliminated the snapping, first we elongated perpendicular incision posteriorly. The incision extended between muscular walls of GM until the only red tissue of GM belly is left (purple line in Fig. 2). It is crucial that the incision should be limited only to “white” fibrous structures of the GM fascial complex. At this point, a presence of snapping was assessed again. If it was absent, the surgery was stopped. If not, an additional oblique incision was made using the radiofrequency probe. It was directed posterosuperiorly at a 45° angle, starting again at the most contracted fascial part of the GMC (orange line in Fig. 2). The presence of snapping was reassessed and if necessary, the next oblique incision was made analogously in the posteroinferior direction (red line in Fig. 2). The snapping was reassessed once again. If it was eliminated after any of the previous three incisions: elongated perpendicular one, posterosuperior or posteroinferior ones (respectively purple, orange, or red lines in Fig. 2), the tissues responsible for ESHS in these particular patients were considered to be the ITB and fascial part of the GMC. In cases in which those elongated perpendicular and additional oblique incisions did not eliminate the snapping, a partial release of the proximal part of GM femoral insertion was performed (brown line in Fig. 2). In such cases all three: ITB, fascial part of GMC, and GM femoral insertion, were considered to be the tissues involved in the ESHS aetiology.

### Rehabilitation

Patients were permitted to perform hip flexion above 90° from the first day post-op. They were instructed to lay on their non-operated side with their legs adducted, slowly flexing the operative hip to >90° every two hours. Guided physiotherapy performed weekly was started two weeks postoperatively. It focused on gentle stretching of released tissues, in order to avoid from healing in a contracted position and on muscular stabilisation of the hip. For the first two weeks, patients were limited to walking in a slight hip abduction and only with crutches but were permitted full weightbearing as tolerated.

### Follow-up assessment

At six weeks post-op, the patients were tested for the presence of snapping, pain when laying on the operated side, ROM, and muscular strength. In order to find potential asymmetries, ROM was assessed in all directions and muscular strength was assessed for hip extension, flexion, and abduction. The above described examination was repeated at the final follow-up of a minimum of 24 months (24-56 months). Additionally, the Multicenter Arthroscopy of the Hip Outcome Research Network (MAHORN) Hip Outcome Tool (MHOT-14) (Additional file 1) was collected preoperatively and at final follow-up.

### Statistical analysis

The nominal variables (tissues involved in the snapping, recurrence, complications) were summarized and presented as percentages and raw values. The continuous variable (MHOT-14 score) was assessed for normality of distribution by the means of Shapiro-Wilk test and afterwards the Student t-test for paired variables was used. Statistical significance was set at standard *p*-value < 0.05.

## Results

In seven out of the 31 hips (22.6%), the ITB was the only tissue responsible for the occurrence of symptoms. In 22 cases (70.9%), the ITB and fascial part of GMC was involved, while in two cases (6.5%), contracture of the GM femoral insertion was also a part of ESHS aetiological origin (Table 1).

**Table 1:** Structures involved in external snapping hip syndrome (ESHS)

Involved structures	Number of patients (%)
Iliotibial band only	7 (22.6)
Gluteal fascia + iliotibial band	22 (70.9)
Gluteal fascia + iliotibial band + proximal part of gluteus maximus femoral insertion	2 (6.5)

There were no recurrences of snapping at six weeks post-op and at final follow-up. There were no reported cases of pain when laying on the operated side and all patients exhibited full symmetrical ROM and muscular strength in Lovett scale. All patients were able to return to their desired activity at an average of eight weeks after the operation. All four patients with bilateral ESHS decided to undergo a surgery on the second hip. The average overall MHOT-14 score increased from a pre-operative score of 46 to 93 at follow-up (*p*<0.001) (Table 2).

**Table 2:** Patient results of the MAHORN Hip Outcome Tool (MHOT-14)

	Pre-operative score	Post-operative score
Symptoms and functional limitations	42	93^*^
Sports and recreational activities	54	91^*^
Job related concerns	65	96^*^
Social, Emotional and Lifestyle Concerns	37	94^*^
Overall	46	93^*^

^*^ *p* < 0.001

Post-operative complications included five cases of asymmetry of the buttocks (16.1%), four cases of haematomas across two patients with bilateral ESHS (12.9%), one case of temporary numbness and pain upon palpation over the trochanteric area (3.2%), and one case of transient asymmetry of the pelvis that presented at two weeks post-op but subsided by six weeks post-op (3.2%).

## Discussion

The most important findings of this study were that the: 1) distribution of ESHS aetiological origin in the patients with possibility to manually reproduce the snapping was identified and 2) in cases where isolated ITB release did not provide intra-operative relief of snapping, extension of the procedure to include a stepwise, tailor-made endoscopic release is needed. Release of involved fascial structures of the GMC up to the proximal part of GM femoral insertion resulted in 0% recurrence rate and notable improvement in all domains of the MHOT-14 score. This confirmed the study hypothesis that intraoperative identification and gradual addressing of all known reasons of ESHS would allow for safe elimination of snapping in all patients.

The clinical relevance of identification of the origin of ESHS is that it allows to directly target the anatomical structure responsible for the occurrence of ESHS symptoms, with maximum preservation of surrounding tissue during surgery. The results of our study are largely consistent to those of Kim et al. [3], who proposed an aetiological classification of ESHS into two types. In type A, the snapping was caused mainly by a contracted ITB, and friction occured over the central lateral aspect of the greater trochanter. In type B, the pathology was more posteriorly as the GMC was mainly involved, and the friction occurred over the posterolateral trochanter. According to our observations, the more flexion necessary to induce snapping, the more posterior the area the greatest contracture was found. For example, patients who demonstrated snapping at relatively small angles of hip flexion (less than 30°), were more likely to have their symptoms resolved after isolated ITB release procedure. These patients are likely to correspond to type A (22.6% of patients in this report, as shown in Table 1). In contrast, patients in whom snapping occurred at greater angles of hip flexion (almost 90°), underwent the subsequent release of the fascial part of the GMC due to persistence of snapping upon intra-operative testing. These patients are likely to correspond to type B (70.9% of patients in this report, as shown in Table 1).. In addition, possibly a type C or a clinical subtype of the type B could be proposed, because in the two cases (6.5%) in which the snapping occurred almost in full flexion near to the chest, it was necessary not only to release the ITB and fascial part of the GMC but also to partially release the GM femoral insertion. This percent was comparable to the 5.2% of patients who did not achieve “excellent” results as reported by Shrestha et al. [21]. Shrestha et al. reported release of ITB and contractures of GMC, however did not report releasing of the GM femoral insertion. It can be hypothesized that the reason for this outcome may be the unaddressed involvement of GM femoral insertion [21]. As their study is the biggest on the topic with more than 200 included patients, it can be considered a fair representation of different aetiological ESHS types [21]. Although numerous studies have demonstrated relatively successful outcomes (Table 3), this study is among a few that have reported tailoring surgical treatment modality depending on the underlying aetiology of the snapping [7, 19, 20]. Although the specific techniques used by other authors differ from our own (diamond-shape resection + GM tenotomy [20],C-shape release [19], vs “fan-like” release in this study), we promote the idea of gradual stepwise release of the lateral hip structures only as needed to eliminate snapping. This ensures maximum tissue preservation while precisely targeting the anatomical structures directly responsible for the occurrence of symptoms and may be one of the reasons why not a single reoperation was needed in neither this study nor in the two other published case series with gradual stepwise release technique[19, 20].

**Table 3.** Comparison of external snapping hip treatment outcomes from various studies

Study	Year	Number of patients	Surgical technique	Outcome measure(s)	Follow-up (months)	Recurrence (%)
White [10]	2004	15 (3M, 12F)	Open step cut ITB release	–	32.5	2 (13.3)
Provencher [22]	2004	8 (4M, 4F)	Open Z-plasty of ITB	Flexion, extension, abduction, internal rotation, external rotation	22.9 (7–38)	0 (0.0%)
Ilizaliturri [2]	2006	10 (1M, 9F)	Endoscopic diamond-shaped ITB release	WOMAC	25	1 (10.0%)
Nam [23]	2011	7 (4M, 3F)	Open modified Z-plasty of ITB	Abduction of hip at 90° flexion, straight leg raising	84 (44–119)	0 (0.0%)
Polesello [13]	2013	8 (1M, 7F)	Endoscopic GM tendon release	mHHS	32 (22–45)	2 (25.0%)
Zini [11]	2013	15 (3M, 12F)	Endoscopic iliotibial band release	VAS, HHS, Tegner	33.8 (12–84)	0 (0.0%)
Yoon [20]	2014	7 (2M, 5F)	Arthroscopic diamond-shaped ITB release + GM tenotomy	VAS, mHHS	19	0 (0.0%)
Shrestha [21]	2017	248 (99M, 149F)	Arthroscopic ITB + GM complex release	Adduction angle, flexion angle	24	0 (0.0%) 13 (5.2%)^b^
Park [6]	2017	17 (17M, 0F)	Open N-plasty of ITB	VAS, mHHS	8–24	0 (0.0%)
Dai [1]	2018	44 (16M, 28F)	Open GM contracture band release	Maximum hip adduction angle, VAS, HHS	12–24	4 (9.1%)
		48 (18M, 30F)	Arthroscopic GM contracture band release			4 (8.3%)
Thomassen [9]	2019	11 (5M, 6F)	Endoscopic release of ITB + bursectomy	NRS^a^, HHS	15–42	1 (9.1%)
Kim [3]	2020	8 (8M, 0F)	Arthroscopic diamond-shaped ITB release ± gluteal sling release	VAS, mHHS, NAHS	3	1 (12.5%)
Chu [8]	2021	18 (10M, 8F)	Arthroscopic diamond-shaped ITB release	VAS, mHHS	84	0 (0.0%)
Tang [19]	2021	96 (35M, 61F)	Arthroscopic C-shaped release around GT	GDS, VAS	23–36	0 (0.0%)
		89 (34M, 55F)	Arthroscopic GM contracture band release			0 (0.0%)
Randelli [14]	2021	22 (6M, 16F)	Arthroscopic GM tendon release	VAS, mHHS, NAHS	18	0 (0.0%)
This study	2023	31 (4M, 26F)	Endoscopic stepwise “fan-like” release of the ITB ± GM complex	Lovett Scale, MHOT-14	24–56	0 (0.0%)

*M,* male; *F,* female; *ITB,* iliotibial band; *GM,* gluteus maximus; *GT,* greater trochanter; *MHOT-14*, MAHORN hip outcome tool; *WOMAC,* Western Ontario and McMaster Universities osteoarthritis index; *VAS,* visual analogue scale; *mHHS,* modified Harris hip score; *NAHS,* non-arthritic hip score; *GDS,* gluteal muscle contracture disability scale; *NRS,* numerical rating scale

^**a**^ Pain and function were rated by the patients using NRS

^b^ The patients that did not achieve “excellent” ratio

### Limitations

A limitation of this study was that we used an older version of the MAHORN Hip Outcome Tool - MHOT-14 (Additional file 1). The MAHORN group now suggests the routine use of an updated version of the MHOT-14, called the International Hip Outcome Tool (iHOT-12) [24, 25]. At the time of our study, the MHOT-14 was the widely used hip functional assessment scale. MHOT-14 and its shortened version - iHOT-12, are both very comprehensive. These scales not only consider typical physical symptoms, but also emotional and lifestyle problems caused by the hip. The only difference between the two outcome tools is that in the updated iHOT-12, two questions were omitted: “How difficult is it to lay on your affected side?” and “How much difficulty do you have at work because of reduced hip mobility?” (Additional file 1) [24]. However, as to first of the omitted questions, in our clinical experience, most of the patients complained of pain while lying on the involved side or after application of direct compression/pressure to the trochanteric region. We believe that use of the MHOT-14 in this study was appropriate at the time of the data collection and should not hinder the ability to compare the treatment outcomes with other studies. As the MHOT-14 is based on patient’s self-assessment of hip function, it did not directly assess the possibility to perform hip movements without snapping – however recurrence of snapping was assessed as well. Another limitation was that our groups was limited to patients in which it was possible to manually reproduce the snap by a physician. This pre-requisition was necessary in order to perform an intra-operative assessment, allowing us to gradually address all known reasons of ESHS in a stepwise, iterative manner. On the one hand it allowed for maximum preservation of surrounding tissue while also precisely targeting the anatomical structure directly responsible for the occurrence of symptoms. However, on the other hand, there is limited generalizability of results on the patients with ESHS in which the physician cannot manually reproduce a snapping. What is more, the impact of narcosis or spinal anesthesia on muscle tension and the occurrence of snapping remains unclear. Nevertheless, in the presented case series we did not face a resolution of snapping after anesthesia in any of the patients.

## Conclusions

Intraoperative identification and gradual addressing of all known reasons of ESHS allows for maximum preservation of surrounding tissue during surgery while precisely targeting the directly involved structures. Endoscopic stepwise “fan-like” release of the ITB and GMC is an effective, tailor-made treatment option for ESHS regardless of the origin of the snapping in the patients with possibility to manually reproduce the snapping.

### Supplementary Information

ESM 1 **Additional file 1** MAHORN Hip Outcome Tool (MHOT-14)

## Data Availability

Data are availalble on request.
