# Supplementary material for: Gluteal Complex is important in External Snapping Hip: intraoperative identification of syndrome origin and endoscopic stepwise release–a case series
Source: Int Orthop. 2023 Sep 5;48(2):401–8. doi: 10.1007/s00264-023-05961-0 (PMC10799799; doi:10.1007/s00264-023-05961-0)
Supplement: Supplementary file 1 — Additional file 1 MAHORN Hip Outcome Tool (MHOT-14) [file 264_2023_5961_MOESM1_ESM.pdf]

Name: ______________________
Date: ______________________

MAHORN Hip Outcome Tool (MHOT[14])

Quality of Life Questionnaire for Young, Active Patients with Hip Problems

Instructions:

- These questions ask about the problems you may be experiencing in your hip, how these problems affect your life, and the emotions you may feel because of these problems.
- Please answer each question with respect to the current status, function, circumstances and beliefs related to your hip.
- Consider the last **month**.
- The questions are formatted so that you can indicate the severity of the problem by marking the line below the question.

Please note:
Please mark the line with a slash at the point which most closely represents your situation.

- If you put a mark on the far **left,** it means that you **feel you are significantly impaired**.
  For example:

Significantly impaired /—————————————————— No problems at all

- If you put a mark on the far **right**, it means that you **do not think that you have any problems** with your hip.
  For example:

Significantly impaired ——————————————————/ **No problems at all**

If the mark is placed in the middle of the line, this indicates that you are moderately disabled, or in other words, between the extremes of 'significantly impaired' and 'no problems at all'. It is important to put your mark at either end of the line if the extreme descriptions accurately reflect your situation.

If the question asks about something that you do not experience, please mark the option:
☑ **I do not do this action in my activities**, where this is appropriate.

I: SYMPTOMS AND FUNCTIONAL LIMITATIONS

The following questions ask about symptoms that you may experience in your **hip** and about the function of your **hip** with respect to daily activities. Please think about how you have felt most of the time over the past **month** and answer accordingly.

1. **How difficult is it for you to walk long distances?**

   Extremely difficult ________________________________ **Not difficult at all**

2. **How difficult is it for you to get up and down off the floor/ground?**

   Extremely difficult ________________________________ **Not difficult at all**

3. **How difficult is it for you to lie on your affected hip side?**

   Extremely difficult ________________________________ **Not difficult at all**

4. **How much trouble do you have with grinding, catching or clicking in your hip?**

   Severe trouble ________________________________ **No trouble at all**

5. **Overall, how much pain do you have in your hip/groin?**

   Extreme pain ________________________________ **No pain at all**

II: SPORTS AND RECREATIONAL ACTIVITIES

The following questions ask about your **hip** when you participate in sports and recreational activities. Please think about how you have felt most of the time over the past **month** and answer accordingly.

6. How concerned are you about your ability to maintain your desired fitness level?

Extremely concerned ______________________________ **Not concerned at all**

7. How much pain do you experience in your hip after activity?

Extreme pain ______________________________ **No pain at all**

8. How concerned are you about cutting/changing directions during your sport or recreational activities?

☐ **I do not do this action in my activities**

Extremely concerned ______________________________ **Not concerned at all**

III: JOB RELATED CONCERNS

The following questions relate to your **hip** with respect to your work or occupational activities. Please think about how you have felt most of the time over the past **month** and answer accordingly.

☐ **I am retired** (please skip section)
☐ **I do not work for reasons other than my hip condition** (please skip section)

9. How much trouble do you have pushing, pulling, lifting or carrying heavy objects at work?

☐ **I do not do these actions in my work**

Severe trouble ______________________________ **No trouble at all**

10. How much difficulty do you have at work because of reduced hip mobility?

Extreme difficulty ________________________________ **No difficulty at all**

IV: SOCIAL, EMOTIONAL AND LIFESTYLE CONCERNS

The following questions ask about social, emotional and lifestyle concerns that you may feel with respect to your **hip** problem. Please think about how you have felt most of the time over the past **month** and answer accordingly.

11. How much trouble do you have with sexual activity because of your hip?

☐ **This is not relevant to me**

Severe trouble ________________________________ **No trouble at all**

12. How much of a distraction is your hip problem?

Extreme distraction ________________________________ **No distraction at all**

13. How concerned are you about picking up or carrying children because of your hip?

☐ **I do not do this action in my activities**

Extremely concerned ________________________________ **Not concerned at all**

14. How much of the time are you aware of the disability in your hip?

Constantly aware ________________________________ **Not aware at all**

QUESTIONNAIRE COMPLETE!
THANK YOU!

MAHORN Hip Outcome Tool MHOT [14/33]

"Quality of Life Questionnaire for Young, Active Patients with Hip Problems"

Scoring the questionnaire

This is a self administered questionnaire. Every question should be answered by the patients. There are exceptions as follows: questions i.e. #21, #28, #32, and the Job related concerns domain, allow patients to check a box that these items are not part of their activity or relevant. All other questions should be answered.

Each question has a response format which is a visual analogue scale that is meant to be exactly 100 mm in length. Therefore, each question can be answered from 0-100 points or 0-100%. Quality of life is a good thing therefore a higher score is better. A score of 100 would be considered optimal quality of life with respect to a person with a hip problem.

It is very simple to score the questionnaire. Measure each visual analogue line from left to right with a 100 mm ruler. Read the score from 0-100 and right down the number (eg. 22 mm equals a score of 22 etc.). The total score is the sum of all of the answered questions divided by the number of questions answered. For example: If a patient only answered 32 out of the 33 questions the numerator would be the sum of the 32 questions and the denominator would be 32.

It is also possible to obtain separate scores on each of the four domains of the questionnaire. The four domains are:

1. Symptoms and functional limitations
2. Sports and recreational activities
3. Job related concerns
4. Social, Emotional and Lifestyle concerns

The same formula applies to each domain by dividing the total score by the number of questions answered within the domain in question. Experience with other similar questionnaires demonstrates that some patients will be more or less impaired in one domain or the other. For example; a patient who has an office job may not have much impairment in their quality of life with respect to this domain. A professional athlete would likely be the opposite.

The questionnaire should be used at baseline assessment of the patient and at follow up appointments. It is recommended that a minimum of one month has passed between administrations of the Hip-QOL, since patients are asked to reflect their answers to the questions over the past month. It is more likely that changes in quality of life will be measureable at longer time periods such as 2-3 months.
